# Adaptation and Validation of the DSM 5 Youth Anxiety Scale—Part I (YAM-5-I) in Colombian Adolescents

**DOI:** 10.3390/healthcare13080900

**Published:** 2025-04-14

**Authors:** Yenny Salamanca-Camargo, José Antonio Muela-Martínez, Lourdes Espinosa-Fernandez, Mª del Mar Díaz-Castela

**Affiliations:** 1 Shool of Psychology, Universidad Pedagógica y Tecnológica de Colombia, Tunja 150001, Colombia; 2 Departament of Psychology, Lagunillas Campus, Universidad de Jaén, 23071 Jaén, Spain; jmuela@ujaen.es (J.A.M.-M.); lespino@ujaen.es (L.E.-F.); mmdiaz@ujaen.es (M.d.M.D.-C.)

**Keywords:** adolescence, Anxiety Disorders Symptoms, assessment, Youth Anxiety Measure

## Abstract

Background/Objectives: In adolescence, anxiety disorders are the most prevalent, besides being highly comorbid, with a tendency to chronicity and persistence in adulthood; although there are different assessment measures with good psychometric properties, in Colombia there are no instruments that include the new international diagnostic classifications, aspects that may hinder accurate diagnosis and consequent care. This psychometric study aimed to adapt and validate the Anxiety Scale for Adolescents YAM-5, part I. Methods: A review of the items of the instrument was carried out, seeking to identify possible difficulties in the use of terms according to the culture; a sample of 536 adolescents linked to different public and private educational institutions from the five regions of the country was applied. The analysis of the instrument was based on the analysis of its reliability by means of Cronbach’s Alpha Coefficient, the construct validity by means of the Exploratory Factor Analysis using the principal components method, and, finally, the Confirmatory Factor Analysis using the Structural Equations technique. Results: An internal consistency of 0.93 and a structural validity with a construct of five correlated dimensions were identified, which best fitted the data collected. Conclusions: The structure examined provides high reliability and structural validity, highlighting benefits such as being of screening type, its low cost, and application aimed at non-clinical populations from the perspective of Colombian adolescents.

## 1. Introduction

Anxiety disorders tend to be highly comorbid with each other; their distinction requires a detailed analysis of the type of situation feared or avoided, the content of the associated thoughts or beliefs, and even the timing and severity of the symptoms [1]. People with symptoms of anxiety come to the clinic with certain manifestations, such as irritability, anger, nervousness, and somatic symptoms, which are often misinterpreted as resistance, disobedience, or even medical problems. This reveals shortcomings in assessment and diagnosis, leading to unnecessary investigations and delays in interventions [2].

One in seven children and adolescents between the ages of 10 and 19 have a mental disorder worldwide. The most common disorders are anxiety, depression, and conduct disorder [3]. Mental disorders are prevalent in Colombia, often chronic, persistent, and comorbid, with anxiety disorder being the most frequent [4]. Some authors warn that, for all health systems, it has become a growing public health problem that requires the attention of specialized and trained personnel [5,6]. With the publication of the DSM 5 [1], the changes in the classification of anxiety disorder have highlighted the need for updated assessment measures, and their adaptation to the cultural context is also considered of great relevance [7].

Although there are a significant number of anxiety scales for screening anxiety symptoms [8,9,10], in Colombia, there are no updated measures according to the DSM-5 diagnostic criteria [1]. From a healthcare perspective, another limitation is the lack of knowledge about assessment tools and guidelines to guide a correct and timely diagnosis and treatment [11]. In this sense, it is important to consider the changes proposed in the DSM-5 regarding the new classification and the adaptation of some of its diagnostic criteria [12].

In the field of dimensional anxiety scales and their psychometric properties, a plethora of studies, including the Anxiety Scale for Adolescents -YAM-5 [13], have found adequate internal consistency and support for construct validity. These studies have been applied to clinical and non-clinical populations from the Netherlands [14], Germans [15], Portuguese [16,17] and Spanish speakers [18]. These findings highlight the need to continue validation studies and analyze the item’s correlation with dimensions for empirical support of the identified differences regarding the original measures. This study adapted and validated the Anxiety Scale for Adolescents YAM-5, part 1, in a group of Colombian adolescents.

## 2. Materials and Methods

Participants: 536 adolescents linked to different public and private educational institutions in the country (51.5% females and 48.5% males) aged between 12 and 17 years (mean 14.36 standard deviation = 1.66), schooled and from Colombia. The selection of the sample was calculated from a simple random sampling, with a margin of error of 5% and a reliability of 95%, developed in two stages: the first, from a stratified sampling with allocation proportional to size, defining five strata corresponding to each of the regions of the country: Caribbean, Pacific, Andean, Orinoco and Amazon; the second, with a random selection of the participants, according to the distribution of strata, sex and level of schooling.

The sample size was determined using data from the National Population and Housing Census [19]. The sample calculation was performed using R statistical software, version 3.6.0.

Instrument: The Youth Anxiety Measure for the DSM-5, YAM-5-I [13], is a tool designed to assess the spectrum of Anxiety Disorder Symptoms in children and adolescents aged 8 to 18 years. The scale is composed of two related sections. The first section (YAM-5-I) is designed to evaluate the presence of major Anxiety Disorders; the second section (YAM-5-II) measures specific phobias and agoraphobia. For the present study, the first section was used, which consists of 28 items and assesses the presence of anxiety disorders such as Separation Anxiety Disorder (AS, 6 items), Selective Mutism (SM, 4 items), Social Anxiety Disorder (SOC, 6 items), Panic Disorder (PD, 6 items) and Generalized Anxiety Disorder (GAD, 6 items) on a Likert scale of 0 to 4 points (Never,0; Sometimes, 1; Quite often,2; Quite often,3; Always,4). Scores for both the overall scale and the included subscales indicate that the higher the score, the higher the levels of anxiety symptoms. Furthermore, the translation into Spanish was considered in [18].

The Cronbach’s alpha value for the non-clinical sample was 0.93, while for the clinical sample, it was 0.92. In contrast, the internal consistency of the subscales varied, exhibiting Cronbach’s alpha values ranging from 0.76 to 0.85. However, the SM subscale demonstrates an exception, with a value of 0.65 for the non-clinical sample and 0.55 for the clinical sample.

Procedure: This text discusses the process of conducting a study on adolescent emotions in Colombia. The study involved obtaining informed consent from both the adolescents and their parents. Small groups were formed based on the school level to administer the study, and a research assistant was assigned to each educational institution. These research assistants received training on the study’s objectives, such as how to contact the institutions, obtain permissions, and administer the instrument. The study was approved by the academic committee of each school and had ethical approval from both a Spanish and Colombian university. The data collection was conducted as part of a larger project that aimed to validate evaluation measures and examine the relationship between expressed emotion and other psychological variables in Colombian adolescents. The project was registered with the code SGI 3619.

Statistical Analysis: Initially, a frequency analysis was performed to discard values that were within the range of the response options. To determine response behavior, scores were compared by age group and gender; reliability was analyzed using Cronbach’s Alpha Coefficient, and construct validity was examined using the principal components method. Lastly, confirmation was processed. On Windows, the statistical package R version 4.4.3 for Windows was downloaded.

## 3. Results

Table 1 shows the mean and standard deviation for YAM-5-I both in the total score and by factors according to sex and age, noting that there is a wide difference between the ratings by sex. For this reason, the Student *t*-test for difference in means was applied to determine the significance, finding that both for the factors and the total, there are significant differences (*p* < 0.001), being greater in the female sex in three of the four factors of the instrument (SA, SOC, PAN, and GAD). In the case of age, no perceptible distinctions were observed in the scores, so ANOVA tests were performed, which effectively showed that there were no significant differences in the scores.

### 3.1. Reliability

Table 2 presents the Cronbach’s Alpha values for the defined factors [16] and the entire questionnaire, taking into account that they did not constitute the definitive dimensions but would be estimated later by means of Factor Analysis.

Although it was found that the measure, in general, obtained a high value (0.93), showing that in its totality, it has internal consistency, the analysis was carried out independently for each factor, and it was found that in the DM factor, it was less than 0.70; therefore, we proceeded to analyze the four items that made it up and it was found that by eliminating item 20, the score in this factor increased to 0.71, which is considered an acceptable value.

Considering the defined structure [13], the validation in its Spanish version [18], and the results of internal consistency, an exploratory factor analysis (EFA) was proposed because this measure has not been validated in Colombian adolescents based on a principal component analysis with orthogonal rotation (varimax). For the extraction of the number of factors, in addition to the results of previous studies, the Kaiser Meyer Olkin criteria (KMO = 0.95) and Bartlett’s test of sphericity (*p*-value < 2.2 × 10^−16^) were applied, allowing the feasibility of the EFA with five factors to be established.

Table 3 shows the factor loadings of the items included in each factor, noting that item 20 does not exceed the value of 0.30, and for this reason, and considering what was observed in the internal consistency analysis, it is eliminated from the analysis [20].

Examining the results in Table 3 and determining the possible correlations between the items and the factors, the latter were defined as follows: Factor 1 (items 5, 9, 13, 14, 18, 27, 22); Factor 2 (items 1, 2, 6, 11, 24, 25); Factor 3 (items 23, 28, 12, 16, 3, 7); Factor 4 (items 17, 21, 26, 8, 4) and Factor 5 (items 10, 15, 19). The percentage of variance explained by each factor was 14.6%, 13.1%, 8.4%, 7.5%, and 6.8%, respectively; although the number of Factors was maintained, no specific name was assigned to the Factors because no concordance was found about the original test [13].

### 3.2. Construct Validity

From the EFA results, a CFA based on structural equations was performed based on six goodness-of-fit indicators: (1) chi-square divided gradually of freedom (the quotient should be less than 4.0); (2) Bentler’s Comparative fit index (the quotient Considering that, to determine a good fit, the CFI and GFI values should be greater than 0.90 (the higher the value, the better the fit), the RMR and RMSEA value 0.05 (the lower the value, the better the fit), and the AIC value (Table 4).

Following the identification of non-compliance with all goodness-of-fit values, it was decided to test the original structure [13] of five factors, defined as follows: (1) AS (items 1, 6, 10, 15, 19, 24); (2) SM (items 2, 11, 25); (3) SOC (items 3, 7, 12, 16, 23, 28); (4) PAN (items 4, 8, 13, 17, 21, 26); (5) GAD (items 5, 9, 14, 18, 22, 27). The goodness-of-fit indices for this factor structure are presented in Table 5.

**Figure 1:**
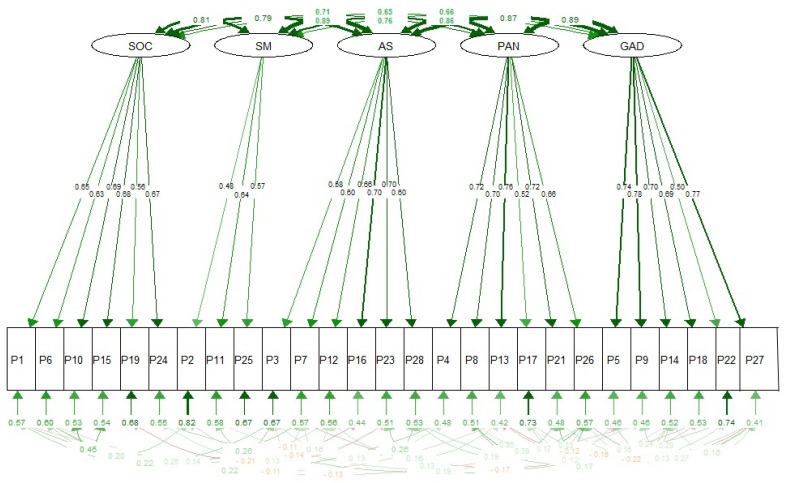
Model on the YAM structure of five correlated factors.

Regarding the goodness-of-fit indices of the three models that were contrasted in Table 4 and Table 5, it was identified that the model with five correlated factors presented better goodness-of-fit values (Figure 1) based on the data collected. This finding supports the conclusions of the original test [13], and it is recommended that item 20 be eliminated for Colombia.

## 4. Discussion

Validation of an instrument is a continuous and dynamic process that leads to greater consistency based on multiple studies of its psychometric properties across cultures, populations, and participants [21]. The validation and adaptation of instruments is an important challenge, as it expands the availability of tests and becomes an input for improving interventions in different areas of psychology [22].

In this study, the original measure was used, but in its translation into Spanish [18], which had the endorsement of its translation by the authors of the instrument, in turn, it was validated and applied in the Spanish-speaking population; finally, the items were reviewed, and it was considered that they were written in a way that was understandable for Colombian adolescents and therefore no additional modifications were necessary.

In Colombia, it is important to conduct more studies to validate the assessment instruments and ensure their validity and reliability. Currently, there is a tendency to use convenience samples located in specific regions, which may not accurately represent the population. Therefore, it is crucial to validate, adapt, and standardize these instruments using samples that are geographically, culturally, socially, and psychologically similar to the intended population [23]. Additionally, there is a need for updated instruments that are based on the diagnostic criteria outlined in DSM-5 [1]. By using these updated diagnostic classifications, it will be easier to understand and assess the prevalence of certain conditions. Overall, more research and attention are needed to develop and utilize assessment instruments that are accurate and effective in the Colombian context.

In this sense, the use of instruments based on the DSM-5 diagnostic criteria as tools for research and clinical practice is considered of great value, supporting aspects such as reliability and diagnostic validity, reducing variability in symptom interpretation, improving communication between professionals, and facilitating the collection of epidemiological data. Additionally, it contributes to more accurate decision-making regarding the choice of treatment or even the comprehensive analysis of a given problem. Specifically, in the case of the YAM-5-I measure, studies conducted in different populations have shown it to be an instrument with adequate internal consistency and support for construct validity [14,15,16,17,18].

When analyzing the structure proposed in the original measure [16] and its translation into Spanish [18], the results of this validation show a high Cronbach’s alpha coefficient (0.94), which is even higher than those reported in adolescents from Netherlands (0.93) [14]; Portuguese (0.91) [16,17]; and Spanish-speaking (0.84) [18]. Additionally, the last two studies highlighted important differences regarding the original test. In the case of the YAM-5-I measure, studies carried out in various populations have shown that it is an instrument with adequate internal consistency and support for construct validity [16,17,18].

Based on the structure proposed in the original measure [16] and its translation into Spanish [17], the findings of this validation reveal a high Cronbach’s alpha coefficient (0.94), even higher than those reported in adolescents from the Netherlands (0.93) [14]; Portuguese (0.91) [17] and Spanish-speaking (0.84) [18]; also highlighting, in the last two studies, important differences regarding the original test.

Regarding the selected sample, we highlight its representativeness derived from the five regions of the country and the inclusion of a stratified sampling according to sex, age, and schooling; additionally, the comparative analysis with findings from other studies in which the same assessment measure was used, together with the review of the items in cultural terms, providing evidence of their psychometric properties. The Spanish translation of the instrument [18], reviewed and approved by the author of the original test [16], and the findings of the study with Spanish-speaking adolescents [18] were decisive for the cultural adaptation since the adjustments were minimal.

In the mean and standard deviation, a significant difference was identified in the assessments by sex, supported by the analysis of the means of the factors and the total score from the Student *t*-test, which was higher in the female sex in the five factors. In the case of age, no discrepancies were found, which were corroborated by the ANOVA tests.

In the case of the Factorial Structure, similarities were identified with the data of the original test [13], with superior goodness for the SM Factor after the elimination of item 20; these findings differ from those reported with the Spanish-speaking population [18] when proposing the elimination of 11 of the 28 items and, in turn, adding one more factor, related to the partitioning of the AS Factor.

Likewise, with the findings of the Portuguese population [18], in which it was determined to eliminate the SM factor, highlighting in this last study a common aspect with the Colombian version regarding the variation in response between men and women as an input for future studies to determine cut-off points by sex, considered as another goodness of the instrument in question.

Although this study did not include a comparative analysis with other anxiety measures, its internal consistency is highlighted, even superior to other scales validated for Colombia, such as the Inventory of State-Trait Anxiety (ISRA), with a Cronbach’s Alpha of 0.70 [8]; the Zung Scale for Anxiety, with 0.77 [9] and the Manifest Anxiety Scale (CMAS-R2), with 0.85 [10]. This analysis was not comparative, as items and their evaluation are based on DSM IV’s diagnostic criteria, and the results are global and not specific to each disorder.

From the findings, it is affirmed that the scale has structural validity with a construct of five correlated dimensions, highlighting advantages such as screening type, low cost, and applicability in clinical and non-clinical populations as a diagnostic and follow-up measure.

As for limitations, although the findings show cultural adaptation and its corresponding validation, it is to continue with studies in clinical populations, from the parent’s perspective, and with comparative analyses with other instruments.

It is recommended to conduct further validation studies in clinical populations to assess the instrument’s effectiveness in diagnosing anxiety disorders in adolescents seeking treatment. Similarly, we need to continue related studies that will allow us to have instruments based on updated diagnostic criteria and, above all, with good psychometric properties to seek greater precision in both clinical and research settings.

## 5. Conclusions

The structure examined provides high reliability and structural validity, highlighting benefits such as being of screening type, its low cost, and application aimed at non-clinical populations from the perspective of Colombian adolescents.

## Figures and Tables

**Table 1:** Mean and standard deviation for the youth anxiety measure for the DSM-5, YAM-5-I by sex and age.

	n	Total M (SD)	GAD M (SD)	PAN M (SD)	SOC M (SD)	SM M (SD)	AS M (SD)
Total	536	33.6 (17.2)	8.8 (4.6)	6.1 (4.4)	6.8 (4.4)	4.8 (2.8)	7.1 (4.5)
Gender							
Female	274	37.19 (15.97)	10.02 (4.48)	7.07 (4.27)	7.17 (4.28)	4.96 (2.76)	7.97 (4.4)
Male	262	29.83 (17.67)	7.50 (4.27)	5.12 (4.29)	6.41 (4.59)	4.58 (2.86)	6.22 (4.46)
Age							
12	97	33.48 (18.18)	7.92 (4.33)	6.05 (4.37)	6.98 (4.64)	5.11 (3.09)	7.42 (4.48)
13	111	36.72 (16.27)	9.05 (4.04)	6.50 (3.91)	7.94 (4.65)	5.18 (2.76)	8.06 (4.24)
14	86	33.13 (17.37)	8.83 (4.78)	6.35 (4.94)	6.24 (4.20)	4.56 (2.83)	7.15 (4.45)
15	91	34.38 (17.59)	9.51 (5.04)	6.23 (4.58)	6.54 (4.06)	4.76 (2.68)	7.35 (4.77)
16	85	31.85 (16.76)	9.07 (4.72)	5.68 (4.06)	6.35 (4.56)	4.44 (2.66)	6.31 (4.28)
17	66	30.27 (16.81)	8.23 (4.38)	5.68 (4.62)	6.29 (4.29)	4.35 (2.78)	5.73 (4.27)

M = Mean, SD = Standard deviation.

**Table 2:** Internal consistency of YAM 5-I.

	Alfa de Cronbach
GAD	0.85
AS	0.84
PAN	0.86
SOC	0.80
SM	0.62
Total	0.93

**Table 3:** Structure matrix (correlations) of the EFA of YAM. The rotation method applied is varimax.

	Factor 1	Factor 2	Factor 3	Factor 4	Factor 5
P1		0.591	0.112	0.108	0.281
P2		0.357	0.144		0.166
P3	0.261	0.566	0.157		
P4	0.531	0.364	0.102	0.306	
P5	0.732	0.122	0.185	0.120	0.181
P6		0.503	0.133	0.166	0.335
P7	0.344	0.452	0.210	0.234	0.113
P8	0.527	0.197		0.560	0.130
P9	0.658	0.151	0.281	0.152	0.129
P10	0.220	0.401			0.696
P11	0.222	0.606		0.114	0.107
P12	0.318	0.450	0.253	0.183	0.210
P13	0.562	0.387		0.271	0.154
P14	0.727		0.196	0.213	0.154
P15	0.287	0.333	0.131		0.684
P16	0.443	0.406	0.428		0.101
P17	0.113	0.261	0.325	0.338	0.262
P18	0.425	0.143	0.483	0.131	0.194
P19	0.167	0.266	0.218	0.278	0.332
P20		0.274	0.246		0.154
P21	0.427	0.252	0.165	0.562	0.353
P22	0.182	0.235	0.391	0.113	
P23	0.328	0.410	0.526	0.119	0.311
P24		0.523	0.306	0.139	
P25	0.197	0.316	0.328	0.260	
P26	0.334	0.147	0.267	0.761	
P27	0.503		0.508	0.330	0.186
P28	0.302	0.348	0.581	0.203	

**Table 4:** Goodness-of-fit indices of the model on the YAM structure based on EFA.

Factorial Model	$\frac{{Χ}^{2}}{gl}$	CFI	GFI	RMR	RMSEA	AIC
Model EFA with correlations	3.039	0.915	0.888	0.05	0.062	34,219.38

**Table 5:** Goodness-of-fit indices of the models on the YAM 5-I structure based on the CFA.

Factorial Model	$\frac{{Χ}^{2}}{gl}$	CFI	GFI	RMR	RMSEA	AIC
Five Factors	3979	0.868	0.84	0.061	0.075	34,534.08
five correlated factors	2212	0.953	0.922	0.048	0.048	33,971.42

## Data Availability

The data presented in this study are available on request from the corresponding author due to (specify the reason for the restriction).
